# Salmagundi

**Published:** 1887-01

**Authors:** 


### SALMAGUNDI.

EDITED BY E. G. BETTY, D.D.S.

---

CAN this be Dr. C. H. Land?

---

IT'S a pity for some that natural gas "ain't good for tooth pullin," for it is "fresh every day" and no mistake.

---

THE first person upon whom the title of doctor in medicine was ever conferrd was William Gordenia. The college at Asti gave the degree in the year 1329.—*Science.*

---

A HEALTHY boy has just been born to an aged couple of St. Joseph, Mo., the father being seventy-one and the mother sixty-five years of age.—*Exchange.*

---

"DOWN IN FRONT" roared a man in the theater, and in response the little lady touched her lofty hat, and her adolescent escort stroked his amateur mustache.—*Daily Paper.*

---

A BOSTON physician thinks he has run across the longest word in use in the English language. He found it in a medical journal, the word being the chemical terminology for cocaine: Methylbenzomethoxyethyltetiahydropyridinecarboxylate. There are fifty-two letters in the word.—*Daily Paper.*

---

SALMAGUNDI begins her fourth year with this number, and has "sweared a swear" that she will try and do better than ever, so that all exchanges will find something worthy of reproduction and put it in italics if they choose.

Our readers must not forget that many little tricks that seem to be of small importance for publication are really valuable and will be thoroughly appreciated by others, so we advise them that this department is open to all such communications.

A COLORED boy at Newbern, N. C., was bitten by a rattlesnake the other day, and there being no doctor accessible, his companions dug a hole in the ground and placed both legs in it up to the hips, packing the mud securely around him. The poison was extracted and the boy is now about well.—*Exchange.*

---

SEVERAL cases of hydrophobia have recently occurred among camels in Algeria. As the animals had never been bitten, the origin of the disease was unaccountable, until it was ascertained that a mad horse had gained admittance to the pasture; and the explanation given by those who studied the case is, that his saliva had fallen on the grass, and the camels had become infected through abrasions in the mouth.—*Clipping.*

---

MESSRS. B. WESTERMAN & CO. announce the continuation of Carus and Engelmann's Bibliotheca Zoologica. The first part of the continuation is expected immediately, and the whole will be completed in 1888. Carus's *Zoologischer Anzeiger* has since 1878 recorded the publications on zoology. The new volume of the Bibliotheca Zoologica is intended to fill the gap between the *Anzeiger* and Carus and Engelmann's Bibliotheca Zoologica, which covered the literature of 1846-60.

---

THE astute Chinese who were discussing civil service reform when our ancestors were building the reed hut and hurling the flint-tipped javelin are said to pay their medical attendants regularly as long as they enjoy good health, but to promptly discontinue their remittances on the first appearance of sickness, to resume only on recovery; which no doubt has arisen from their absurdly attempting to live up to a foolish old proverb of ours about "an ounce of prevention."—*Woods Hutchinson.*

---

A NEW laboratory-burner, devised by a Detroit inventor, appears to be both simple and efficient. The base of the burner is provided with a stationary needle-valve surrounded by a vertically adjustable jet-tube with a conical aperture controlled by the valve. Arms extending upward from the jet-tube support a vertically adjustable mixing-tube, constructed so as to close the upper end of the jet-tube when desired. The proportions of gas and air, as well as the size of flame, may be regulated by a simple rotation of the jet-tube upon the base.—*Science.*

---

EUGENOL.—Dr. Garret (Newkirk), recently wrote a short article for the *Cosmos* on the above subject, reproduced in *The Dental Eclectic* for November, in which he says: "I do not remember to have seen any where a fully appreciative notice of *Eugenol* among the list of drugs used as disinfectants, antiseptics," etc. If he had attended the meeting of the American Dental Association at Saratoga about three years ago, he would have heard Dr. A. W. Harlan read a paper upon it, and again at the Mississippi Valley Association two years ago, both of which, including the discussions, were published in the REGISTER.

---

THE dentists of Cleveland have recently organized a Society that bids fair to be a good one. The officers are: President, D. R. Jennings; Vice-President, John Stephan; Secretary, P. H. Keese; Treasurer, S. B. Dewey. A private letter to us has this to say regarding it:

"After considerable labor in 'borning' we have brought forth a quite healthy infant and christened it as above. We start out with a membership of fourteen of our leading operators, and we shall spare no effort to make it a success.

Our meetings are to be held on the first Saturday of each month, and we are to have a paper at each meeting from each member in turn, who shall announce the subject of his paper at the meeting prior to the reading of the same. When the subject has been announced the President appoints two members whose duty it shall be to open the discussions. So you see that we shall have at least three persons who will come prepared to handle the subject intelligibly if not intelligently."

This "practical" feature is a hustler and will be the means of always providing material of interest and might with great advantage be adopted by other Societies.

Correction.—On page 607, December number of the Register, in our report of the O. S. D. S., Dr. Harlan is credited with saying: "It was also found that a tooth immersed in pure sulphuric acid for a week showed no effects of the acid upon the enamel," whereas the doctor says he said Herbst's *Obtundent.* It is very likely Dr. Harlan meant the obtundent, though he said sulphuric acid, as Dr. Taft's remarks on page 608 amply evidence. In the heat of a debate speakers often say things that don't look right in print, and the reporter's notes made at the time, are more to be relied upon than the memory of the speaker.

---

The following startling advertisement appeared in the Elkton (Ky.) *Banner*, January 11th, 1856:

Horrid Murder.—I tell you what is a fact; I wish people would pay me all they owe me, so that I could pay a part of what I owe!! Some of you have owed me a long time—you have! So come along and fork up! I have practiced twenty years, and have rarely called on a man for money.

I think I know nearly all about saving teeth and all necessary operations on them, such as plugging, scaling, extracting, and inserting on pivot or gold plate. I promise in future more promptness, better work (if possible), higher charges (if any difference), more dunning, more frequent settlements, and more thanks if my former liberal patronage be continued. My work is warranted as long as a Christian could ask it. I extract teeth gratis for those who are too poor or too ugly to pay for it.

---

Dr. Williams, in the *St. Louis Medical and Surgical Journal*, relates an interesting case of temporary blindness from the excessive use of tobacco. The patient was a blacksmith thirty-two years of age, who complained of failure of vision to such an extent that he could no longer see to drive nails in shoeing, and was compelled to depend on his sense of feeling. His health was good, he having no other complaint. Vision was found to be only one-sixth of what it should be. Things appeared to him to be covered with a dense mist. For many years he had been an excessive smoker, using the strongest tobacco to be had. Tobacco amaurosis is quite common, but usually in men beyond middle life. The probability of recovery is great if the habit is given up, and this should be done gradually. In this case, a very few days' abstinence from tobacco caused an improvement in vision, and the man has now made material progress toward recovery. Dr. Williams does not regard chewing tobacco as so likely to produce this defective vision as the habit of smoking.

---

Soap as a Civilizer.—The introduction of soap, it is said, is doing much to civilize the people of the Holy Land. A large soap factory has been established on the site of the ancient Shechem, and the people are beginning to use it on their persons instead of trying to eat it as they did at first. Along with the introduction of soap other reforms are going on. Bethlehem has been rebuilt, and the streets are lighted with gas. Ceseræa is having a building boom. Nazareth is becoming the headquarters of big olive-oil speculators.

Corner lots in Joppa are going up with a rush, and real estate in Mount Carmel is largely held by speculators for an advance. All around Shechem there is a lively demand for good soap fat, and the sleepy inhabitants of Ramoth Gilead think of building a glue factory. Jerusalem is waking up also. It has a street-cleaning bureau, big clocks on its public buildings, and its suburbs are being built up rapidly. Even in the Vale of Gehenna the price of land has gone up. The ladies of Jerusalem take all the Parisian fashion journals and know all about the latest style of hair-dressing.—*N. Y. Tribune.*

---

Mississippi Valley Association of Dental Surgeons.—The next annual meeting of this time-honored Society is near at hand, and it becomes us all to make preparations for it. The intention of the officers is to make it the very largest in point of numbers in attendance, and the very best in interest. The papers will be numerous and upon a variety of subjects for the reason that a gold medal is offered for the best essay, the choice of subject to be at the discretion of the essayist.

This, of course, offers the very widest field, and will make a programme at once unique and valuable. All those who have papers prepared, or will have them sometime during the present month, will confer a favor upon the Executive Committee by notifying the Chairman, Dr. N. S. Hoff, No. 264 Race street, Cincinnati, or the writer hereof, the name of topic of course being given. If this is done promptly it will enable the committee to make up the programme, at least approximately, and so notify the members through the journals and by circular as well. Gentlemen in Cleveland, Toledo, Chicago, and Cincinnati have promised papers, and we shall no doubt hear from Pittsburgh, Louisville, Lexington, St. Louis, Nashville, New Orleans, Indianapolis, Xenia, and other places. If every member will come and bring a new member with him, the attendance will be all that can be desired, and the discussions of inestimable value

---

A Chip of the Old Block.—A nice little boy, reared in the intellectual and heterodox atmosphere of Boston, happened to be a witness in a case in Cincinnati, and the question arose as to his being old enough to understand the nature of an oath, so the Judge investigated him.

"Well, Wendall," he said, kindly, "do you know where bad little boys will go when they die?"

"No, sir," replied the boy with confidence.

"Goodness gracious!" exclaimed the Judge in shocked surprise; "don't you know they will go to hell!"

"No, sir. Do you!"

"Of course I do."

"How do you know it?"

"The Bible says so."

"Is it true?"

"Certainly it is."

"Can you prove it?"

"No, not positively; but we take it on faith," explained the Judge.

"Do you accept that kind of testimony in this Court?" inquired the boy, cooly.

But the Judge didn't answer; he held up his hands and begged the lawyers to take the witness.—*Washington Critic.*

